# The performance of large language models in dentomaxillofacial radiology: a systematic review

**DOI:** 10.1093/dmfr/twaf060

**Published:** 2025-08-12

**Authors:** Zekai Liu, Andrew Nalley, Jing Hao, Qi Yong H Ai, Andy Wai Kan Yeung, Ray Tanaka, Kuo Feng Hung

**Affiliations:** Oral and Maxillofacial Radiology, Division of Applied Oral Sciences and Community Dental Care, Faculty of Dentistry, The University of Hong Kong, Hong Kong SAR, 999077, China; Oral and Maxillofacial Radiology, Division of Applied Oral Sciences and Community Dental Care, Faculty of Dentistry, The University of Hong Kong, Hong Kong SAR, 999077, China; Oral and Maxillofacial Radiology, Division of Applied Oral Sciences and Community Dental Care, Faculty of Dentistry, The University of Hong Kong, Hong Kong SAR, 999077, China; Department of Diagnostic Radiology, Li Ka Shing Faculty of Medicine, The University of Hong Kong, Hong Kong SAR, 999077, China; Oral and Maxillofacial Radiology, Division of Applied Oral Sciences and Community Dental Care, Faculty of Dentistry, The University of Hong Kong, Hong Kong SAR, 999077, China; Oral and Maxillofacial Radiology, Division of Applied Oral Sciences and Community Dental Care, Faculty of Dentistry, The University of Hong Kong, Hong Kong SAR, 999077, China; Oral and Maxillofacial Radiology, Division of Applied Oral Sciences and Community Dental Care, Faculty of Dentistry, The University of Hong Kong, Hong Kong SAR, 999077, China

**Keywords:** generative artificial intelligence, large language models, chatbots, dento-maxillofacial radiology

## Abstract

**Objectives:**

This study aimed to systematically review the current performance of large language models (LLMs) in dento-maxillofacial radiology (DMFR).

**Methods:**

Five electronic databases were used to identify studies that developed, fine-tuned, or evaluated LLMs for DMFR-related tasks. Data extracted included study purpose, LLM type, images/text source, applied language, dataset characteristics, input and output, performance outcomes, evaluation methods, and reference standards. Customized assessment criteria adapted from the TRIPOD-LLM reporting guideline were used to evaluate the risk-of-bias in the included studies specifically regarding the clarity of dataset origin, the robustness of performance evaluation methods, and the validity of the reference standards.

**Results:**

The initial search yielded 1621 titles, and 19 studies were included. These studies investigated the use of LLMs for tasks including the production and answering of DMFR-related qualification exams and educational questions (*n* = 8), diagnosis and treatment recommendations (*n* = 7), and radiology report generation and patient communication (*n* = 4). LLMs demonstrated varied performance in diagnosing dental conditions, with accuracy ranging from 37% to 92.5% and expert ratings for differential diagnosis and treatment planning between 3.6 and 4.7 on a 5-point scale. For DMFR-related qualification exams and board-style questions, LLMs achieved correctness rates between 33.3% and 86.1%. Automated radiology report generation showed moderate performance with accuracy ranging from 70.4% to 81.3%.

**Conclusions:**

LLMs demonstrate promising potential in DMFR, particularly for diagnostic, educational, and report generation tasks. However, their current accuracy, completeness, and consistency remain variable. Further development, validation, and standardization are needed before LLMs can be reliably integrated as supportive tools in clinical workflows and educational settings.

## Introduction

Large language models (LLMs) are types of generative artificial intelligence (Gen-AI) models that are trained on large amounts of text data and can generate human-like text based on the input provided.^1^ These models can execute complex tasks such as answering questions and providing recommendations. AI chatbots are applications designed based on LLMs to comprehend user inputs, process information, and generate suitable responses. In recent years, publicly accessible AI chatbots, such as ChatGPT, Bing, and DeepSeek, have been increasingly integrated into daily life. Additionally, specific industries have developed task-oriented, in-house LLMs to enhance efficiency in areas such as customer service and other specialized fields.

The potential of LLMs and AI chatbots in healthcare has been increasingly investigated. The promising results of LLMs and AI chatbots in healthcare have advanced their development and application across various medical specialties, including radiology. A cloud‐based intelligent system developed based on LLMs has been introduced to assist patients in seeking medical care and registration.^2^ This system can predict possible disease categories based on patients’ histories of presenting complaints and recommend the appropriate medical subspecialty for patients when scheduling a doctor’s appointment. Previous studies have reported that some AI chatbots performed similarly to clinicians in medical licensing examinations and specialized radiology assessments.^3^^,^^4^ Certain LLMs have been specifically designed to assist radiologists in formulating reports.^5^^,^^6^

Previous studies indicate that a commercially available AI chatbot, ChatGPT, can effectively simplify radiology reports without compromising essential details about the case, which can help patients to comprehend their situations better.^7^ More recently, there has been a surge in the development of innovative vision-language models in radiology. These models can integrate understanding from both image and text inputs to process and merge information from both modalities, performing tasks such as generating image captions, interpreting radiology reports, assisting in diagnostic procedures, and enhancing patient-doctor communication regarding radiological images and findings.^8^

Currently, studies have been conducted to develop new LLMs, substantially fine-tune existing LLMs, or evaluate the performance of existing AI chatbots for specific tasks in the field of radiology. However, the current performance of LLMs, specifically for dento-maxillofacial radiology (DMFR)-related tasks, has not been systematically investigated. Therefore, this study aimed to systematically review the current performance of LLMs in tasks related to DMFR.

## Methods

This systematic review was reported following the Preferred Reporting Items for Systematic Reviews and Meta-Analyses (PRISMA) guideline.^9^ The study protocol was registered with the International Prospective Register of Systematic Reviews, PROSPERO (registration code: CRD42024617397).

### Search strategy and selection process

A systematic search was conducted in June 2025 via 5 electronic literature databases, including PubMed, Medline (Ovid), Embase (Ovid), Web of Science, and Scopus. The search strategy was constructed using 2 main concept groups, each consisting of relevant synonyms and related terms combined with the Boolean operator OR. Group 1 comprised AI, LLM, and chatbot-related terms (ie, “natural language processing,” “NLP,” “language model,” “chatbot,” “ChatGPT,” “GPT,” “BARD,” “BERT,” and “BING”). Group 2 comprised DMFR-related terms (ie, “dentomaxillofacial radiology,” “oral radiology,” “dental radiology,” “DMFR,” “OMFR,” “cone beam computed tomography,” “CBCT,” “panoramic radiography,” “OPG,” “periapical radiography,” “cephalometric radiography,” and “dental X-ray”). The 2 groups were then combined using the Boolean operator AND to ensure that only articles containing at least 1 term from each group were retrieved. Vocabulary and syntax were adapted as appropriate for each database. The full search strategies for all databases are detailed in Table S1. Records were collated in EndNote Version 21 (Clarivate Analytics, Philadelphia, USA). The titles were automatically checked for duplicates. The electronic literature database search was conducted without restrictions on publication period.

The study selection process consisted of 2 stages. In the first stage, 2 postgraduate students in DMFR (Z.L. and J.H.) independently screened the titles and abstracts of all identified records. Records were retained for full-text assessment if their titles and abstracts contained information likely relevant to both DMFR and LLMs, specifically studies involving the use of an AI, LLM, or chatbot tool to generate text output in response to DMFR-related text or image inputs. Records indicating that the articles were not published in English were excluded at this stage. When the relevance of a record could not be determined from the title and abstract, the record was retained for full-text evaluation. In the second stage, the full texts of the selected records were retrieved and assessed independently by the same reviewers to confirm final eligibility. Articles that could not be accessed in full-text were excluded. Review articles, editorials, correspondences, and letters to the editor relevant to both DMFR and LLM were screened solely for potentially eligible original studies in their reference lists, but these article types were not included in this systematic review. Only peer-reviewed original studies that met the following inclusion criteria were included:

1. Studies reporting the initial development, fine-tuning, or validation of a language model applied to texts or images in the field of DMFR; and
2. Studies reporting evaluation metrics that indicate the correctness of the output generated by the language model.

Any discrepancies between reviewers at either stage were resolved through discussion with a board-certified oral radiologist (A.N.) and a faculty member experienced in dental AI research (K.F.H.). Additionally, the reference lists of the included studies were manually examined to identify any further eligible studies.

### Data extraction

Data extraction process was conducted by a postgraduate student in DMFR (Z.L.) and a board-certified oral radiologist (A.N.) using a standardized template created in an Excel spreadsheet (Microsoft Corporation, Redmond, Washington). Data items included author, year of publication, the purpose and design of the study, type of LLM, source of the images or texts, language applied to the LLM, dataset, the input and output, model performance, evaluation methods, and reference standard. Any uncertainties during data extraction were resolved through discussion with the third reviewer (K.F.H.).

### Quality analysis

The methodological quality of the included studies was assessed by 3 reviewers including a postgraduate dental student in DMFR (Z.L.), a board-certified oral radiologist (A.N.), and a faculty member experienced in dental AI research (K.F.H.). The customized assessment criteria (Table 1), adapted from the 2025 TRIPOD-LLM reporting guideline for studies using LLMs,^10^ were used to evaluate the methodological quality of the included studies with a specific focus on the risk of bias in 3 major domains (dataset, model performance evaluation, and reference standard). These domains were selected to address 3 major questions that could affect the reliability of results in studies involving LLMs, which are (1) “Were the source and characteristics of the dataset used for developing, fine-tuning, or testing the LLM clearly described?”; (2) “Were the quality and accuracy of the LLM’s generative outputs, clearly assessed using reliable methods to identify any false or misleading responses?”; and (3) “Was the ground truth/reference standard used to evaluate the accuracy of the LLM’s outputs sufficiently valid and reliable? (eg, established by multiple assessors with specified qualifications).” Studies were rated on a 3-point scale as low, high, or unclear, reflecting potential bias concerns in each domain. Studies that reported all required items in a given domain were assigned a “low” risk-of-bias rating for that domain. If any required items were missing, the rating was classified as “high.” For studies that reported all required items but provided insufficiently clear descriptions, the risk of bias in that domain was categorized as “unclear.”

**Table 1.** Customized assessment criteria, adapted from the 2025 TRIPOD-LLM reporting guideline, used to evaluate the methodological quality of the included studies with a specific focus on the risk of bias in 3 major domains: dataset, model performance evaluation, and reference standard.

Risk of bias		
Dataset	Performance evaluation	Reference standard
For datasets containing only text, the following information should be provided: The source and language of the texts Information indicating when the text was created A detailed description of how the texts were obtained For datasets containing only images, the following information should be provided: The source of the images A detailed description of how the images were selected For datasets containing both text and images, all of the above information should be provided.	Specify how the LLM’s performance was compared to other LLMs, human observers, or established standards. If outcome assessment involves subjective interpretation, the following requirements should be met: The qualifications of the assessors should be reported. Provide a detailed description of how the quality and accuracy of generative outputs were assessed.	If the reference standard (ground truth) was determined based on subjective interpretation, the following requirements should be met: Multiple assessors including at least 1 radiologist should be involved. The qualifications of the assessors should be reported.

Abbreviation: LLM = large language model.

## Results

**Figure 1.**
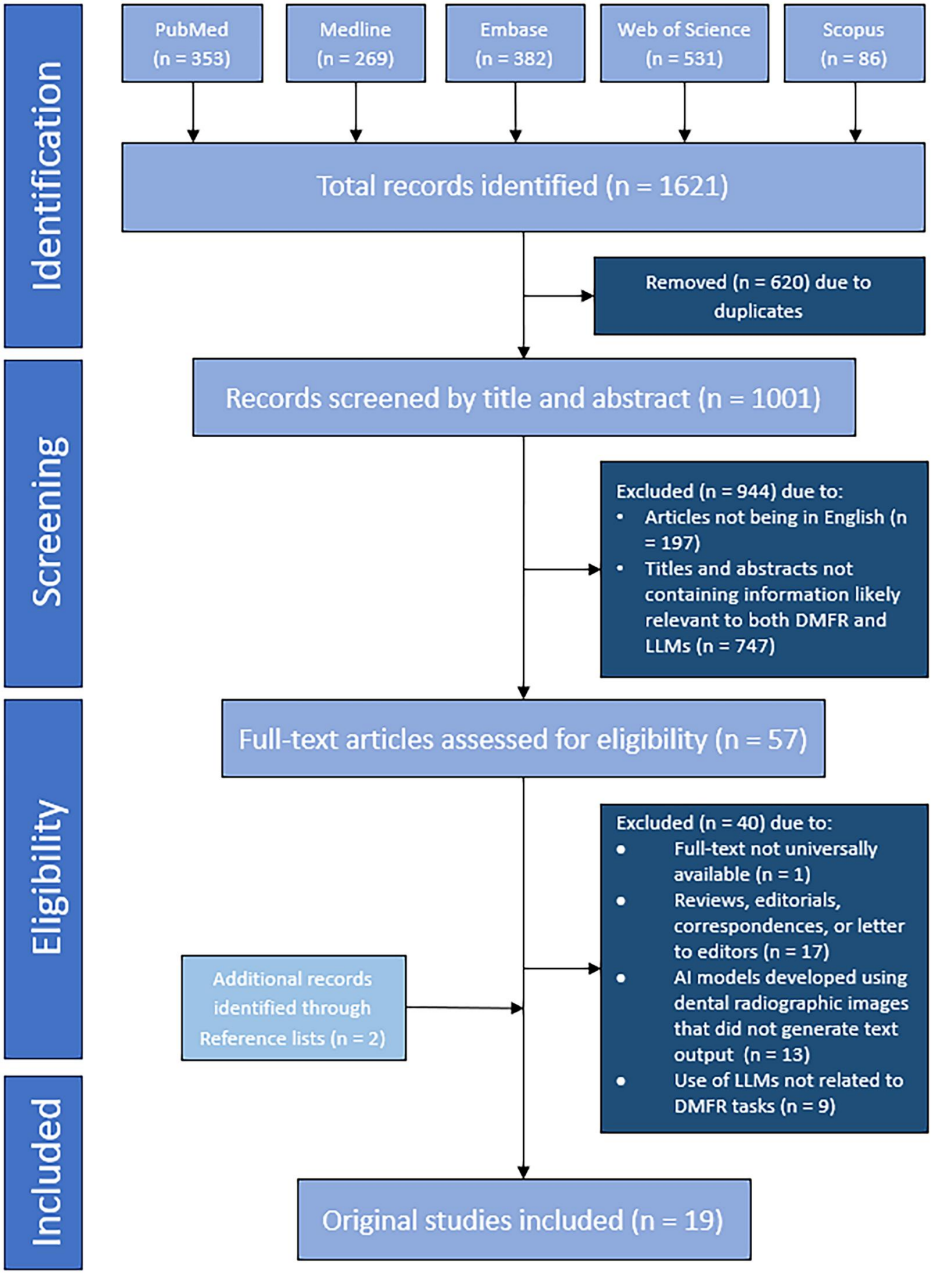
Flowchart of the study selection process.

A total of 1621 records were identified through the search. After removing 620 duplicates, 1001 records remained for title and abstract screening. Subsequently, 57 articles were selected for full-text review. Forty articles were excluded due to full text not universally available (*n* = 1), article types other than original studies (*n* = 17), AI models developed using dental radiographic images that did not generate text output (*n* = 13), and use of LLMs not related to DMFR tasks (*n* = 9). Additionally, 2 eligible studies were identified through reference checking. Therefore, a total of 19 original studies were included in this systematic review. The PRISMA flowchart illustrates the study selection process in Figure 1.

The 19 included studies were published between 2023 and 2025 (Table 2). These studies investigated the use of LLMs for tasks including the production and answering of DMFR-related qualification exams and educational questions (*n* = 8),^11–18^ LLM-assisted diagnosis and treatment recommendations (*n* = 7),^19–25^ and radiology report generation and patient communication (*n* = 4).^26–29^ Regarding input modalities, 9 studies used text-only input (eg, clinical scenarios, multiple-choice questions [MCQs], short asked questions), 5 studies used image-only input (eg, panoramic or periapical radiographs), and 5 studies incorporated both text and image input.

**Table 2.** Overview of the 19 studies included in this systematic review.

Author	Purpose of the study	Study design	LLM	Text or image input	Language of the texts	Source of the texts or images	Model output	Code and data availability	Conclusion
DMFR-related qualification exams and educational questions									
Jeong et al (2024)^13^	To evaluate the performance of 4 LLM-based chatbots compared to dental students in a DMFR examination	Comparative study using 52 exam questions, analysed by educational content and question type	GPT-3.5, GPT Plus (GPT-4), Bard (LaMDA/PaLM2), Bing Chat (GPT-4 based Prometheus)	Text input only: multiple-choice and short-answer questions	Korean	Regular oral and maxillofacial radiology examinations from Yonsei University College of Dentistry, April and June 2023	MCQs: a single correct answer from several options SAQs: key conceptual terms	Supplementary material is available at *Dentomaxillofacial Radiology* online.	Chatbots performed unsatisfactorily compared to students, especially in image interpretation. Further training with domain-specific data and validation of responses are needed.
Morishita et al (2024)^15^	To assess the capabilities of GPT-4V with image recognition in answering image-based questions from the Japanese National Dental Examination	Study using a retrospective dataset of exam questions, comparing GPT-4V’s answers to the official correct answers	GPT-4V (GPT-4 with vision, OpenAI, September 25, 2023 version)	Both text (exam questions) and images (CT, MRI, X-rays, intraoral/extraoral photographs, diagrams, tables) from the exam	Japanese	116th Japanese National Dental Examination, January 2023, Ministry of Health, Labour and Welfare of Japan	One correct choice among 5 provided choices, rationale for the correct and incorrect choices in Japanese	https://www.mhlw.go.jp/seisakunitsuite/bunya/kenkou_iryou/iryou/topics/tp230524-02.html	The version (Nov 2023) of GPT-4V exhibited significant limitations in handling image-intensive and complex clinical dental questions and is not yet suitable as an educational support tool for dental students.
Mohammad-Rahimi et al (2024)^14^	To evaluate the performance of 6 AI chatbots on controversial and difficult questions in oral pathology, oral medicine, and oral radiology, including the quality and validity of their citations	Comparative evaluation study using expert panel assessment of chatbot responses to a standardized set of questions	GPT-3.5, GPT-4 (OpenAI), Bing (Microsoft), Google Bard, Claude (Anthropic), Sage (Poe)	Text input only: 44 questions in oral pathology/medicine and 22 in oral radiology, including controversial, basic, and 1 trick question	English	Expert-generated and AI-generated questions based on clinical controversies and practice	Textual answers to each question, with citations provided when prompted	Questions are available in Supplementary material associated with this article can be found in the online version at doi: 10.1016/j. oooo.2024.01.015.	GPT-4 was the most reliable chatbot for controversial dental topics, but fabricated citations were common across chatbots.
Hak-Sun and Gyu-Tae (2025)^11^	To evaluate and compare the quality of national dental board-style examination questions generated by a LLM and by human experts	Prospective cross-sectional study with item analysis of examination questions answered by senior dental students	GPT-4o (OpenAI)	Text input: MCQs generated from DMFR textbook content	Korean	Questions generated from 22 chapters of the content of a most frequently used DMFR textbook in Korea	Multiple-choice, board-style examination questions with 5 options and 1 correct answer	N/A	ChatGPT can generate dental board-style exam questions of equivalent quality to those written by human experts, but hallucinations require human oversight.
Turunç Oğuzman and Yurdabakan (2024)^18^	To compare the performance of GPT-3.5 and Bard through multiple-choice dental specialty entrance examination questions	Comparative study using exam question to assess the correct answer rates between LLMs	GPT-3.5 (OpenAI) and Bard (Google)	Text input only: multiple-choice questions in prosthodontics and DMFR	Turkish	Official Dental Specialty Entrance Examination questions from the Turkish national database (ÖSYM), up to 2021	One correct answer to the question	N/A	These LLMs are not yet reliable educational tools but could be used as a supplement to traditional educational methods.
Tassoker (2025)^17^	To evaluate and compare the performance of GPT-3.5, GPT-4 Omni (4o), Google Bard, and Microsoft Copilot in answering multiple-choice DMFR questions from dental specialty admission exam in Turkey	Comparative study using 123 text-based, DMFR MCQs with responses assessed for accuracy, word count, and response time	GPT-3.5, GPT-4 Omni (4o), Google Bard, and Microsoft Copilot	Text input only: MCQs	Turkish	Open-source question bank of the Turkish Dental Specialty Admission Exam 2012-2021 (https://www.osym.gov.tr/TR , 15070/dus-cikmis-sorular.html)	One correct answer to the question and responses analysed for word count and time	N/A	ChatGPT-4o had the highest accuracy (86.1%) and advanced reasoning capabilities, making it a promising educational tool in DMFR.
Russe et al (2024)^16^	To develop a content-aware chatbot based on GPT-3.5-Turbo and GPT-4 specialized on the German S2 CBCT dental imaging guideline and compare performance to humans	Comparative study evaluating chatbot and human performance on 40 guideline-based questions	GPT-3.5-Turbo and GPT-4 (OpenAI)	Text input only: questions regarding the use of CBCT	German	German S2 Guideline for CBCT in dental imaging (official guideline document)	Answer (Yes or No) and explanations to the question	Code available on GitHub (https://github.com/maxrusse/CBCTChat); data available from the corresponding author by request	A content-aware GPT-4 chatbot provided reliable, accurate, and trustworthy recommendations at expert practitioner level, outperforming GPT-3.5-Turbo and early-career practitioners.
Helvacioglu-Yigit et al (2025)^12^	To compare the quality and readability of the responses generated by 3 AI chatbots in answering frequently asked questions related to DMFR to assess their suitability for patient education	Comparative study using 15 DMFR-related FAQs posed to 3 AI chatbots and evaluated by 3 DMFR specialists	GPT-3.5 (OpenAI), Gemini 1.5 Pro (Google), Copilot (Microsoft)	Text input: 15 DMFR-related FAQs	English	FAQs selected from Health Physics Society, American Academy of Oral and Maxillofacial Radiology, and Royal College of Dental Surgeons of Ontario professional websites	Text-based responses to each FAQ	N/A	Although chatbots are relatively good at responding to FAQs, validating AI-generated information using input from healthcare professionals can enhance patient care and safety.
Diagnosis of dental diseases and conditions									
Uranbey et al (2024)^25^	To assess the diagnostic accuracy and therapeutic strategies of GPT in comparison to dental professionals across 12 clinical cases	Comparative study using retrospective clinical case scenarios	GPT 3.5 (OpenAI) and Gemini (Google AI)	Text input only: retrospective clinical scenarios (patient history, complaints, lesion characteristics, radiographic features)	Turkish/English	Clinical case data from archives of the Department of Oral and Maxillofacial Surgery, Ordu University, Ordu, Turkey	3 potential differential diagnoses (ranked, with references) and treatment plans for the most likely diagnosis	N/A	ChatGPT demonstrated high accuracy in providing differential diagnoses and acceptable treatment plans, comparable to professionals, but with potential biases and limitations.
Hu et al (2024)^21^	To assess the performance of GPT in generating diagnoses based on chief complaint and CBCT radiologic findings	Retrospective evaluation using 102 CBCT cases	GPT 3.5 (OpenAI)	Text input only: chief complaint and CBCT radiologic findings	English	CBCT reports and radiologic findings from Nanjing Stomatological Hospital, China	Radiologic impression, itemized analysis, clinical diagnosis, possible pathological diagnosis, and differential diagnoses	The [.xlsx] data and [.docx] used to support the findings of this study were supplied by [Zitong Lin] under license and so cannot be made freely available. Requests for access to these data should be made to [Zitong Lin, E-mail: linzitong_710@163.com]	The model’s performance is variable, depending on the complexity of the task, and professional oversight is still crucial due to a certain degree of error rate.
Silva et al (2024)^24^	To evaluate the performance of GPT in describing radiolucent lesions in panoramic radiographs and establishing differential diagnoses	Comparative study with 28 panoramic radiographs evaluated by 3 expert radiologists and GPT-3.5	GPT-3.5 (OpenAI)	Image input via hyperlink to each panoramic radiograph. Prompts requested structured descriptions and differential diagnoses	English	28 panoramic radiographs from open-access clinical case repositories: radiopaedia.org and CDI Peru	1. Description: internal structure, unilocular or multilocular, periphery, shape, location, and effect on adjacent structures. 2. Impressions: origin, behaviour, and nature. 3. Differential diagnoses	N/A	GPT-3.5 showed variable and limited performance in describing and diagnosing radiolucent lesions on panoramic radiographs, and is not suitable for clinical application at present.
Aşar et al (2025)^20^	To evaluate the accuracy of LLMs in detecting supernumerary teeth on periapical radiographs	Comparative study using 180 periapical radiographs	GPT-4V (OpenAI), GPT-4o (OpenAI), and a customized GPT-4V (CGPT-4V) fine-tuned for supernumerary tooth detection	Image input: periapical radiographs (JPEG, 470 × 620 pixels); standardized prompt: “Are there any supernumerary teeth in the radiograph above?”	English	Radiographs from the Department of Pediatric Dentistry, Faculty of Dentistry, Selçuk University, Turkey; annotated and selected by 3 dental experts	Textual answer indicating presence/absence (and, if present, localization) of supernumerary teeth in each radiograph	Datasets are not publicly available but are available from the corresponding author upon reasonable request	Customized GPT-4V achieved the highest diagnostic accuracy (91%) and lowest false positive rate. Domain-specific customization improves LLM diagnostic performance in dental radiology.
Kahalian et al (2024)^22^	To evaluate the performance of GPT-4.0 in identifying anatomical landmarks, cysts, and tumours in oral and maxillofacial radiographic images, and to assess whether providing diagnostic clues improves accuracy	Comparative study with 52 radiographic images analysed by GPT-4.0 with and without diagnostic clues	GPT-4.0 (OpenAI)	Image input: periapical, panoramic, CBCT images (with and without clues such as location, internal structure, peripheral structure, adjacent structures)	English	52 radiographic images from the archive of the Department of Oral and Maxillofacial Radiology, Health and Technology University in Kocaeli, Turkey, collected May-July 2024	1. Location 2. Internal structure 3. Peripheral structure 4. Adjacent structures	N/A	ChatGPT-4.0 showed a tendency to misdiagnose closely located anatomical structures and by adding additional clues its performance showed improvement, while its ability to recognize diverse differential diagnoses remains limited.
Salmanpour and Akpınar (2025)^23^	To evaluate the diagnostic accuracy of GPT-4.0 in determining the labiolingual position of impacted maxillary canines and detecting resorptive changes in adjacent incisors using panoramic radiographs	Observational study with 105 patient radiographs assessed by GPT-4.0	GPT-4.0 (OpenAI)	Image input: panoramic radiographs of maxillary impacted canines, provided to GPT-4.0 for classification	English	Images from the archive of Afyonkarahisar Health Sciences University, Turkey	Texts describing the canine position (labial, mid-alveolar, palatal) and presence/absence of incisor resorption	Data and materials are available at the Orthodontic Department in the Faculty of Dentistry, University of Afyonkarahisar Health Sciences University	ChatGPT-4.0 demonstrated insufficient accuracy for both tasks and is unsuitable for clinical application in determining canine position or incisor resorption from panoramic radiographs.
Ana et al (2025)^19^	To evaluate the accuracy and reproducibility of GPT-4o in interpreting panoramic radiographs for lower third molar assessment	Observational study on 30 panoramic radiographs with GPT-4o responses generated using a standardized prompt	GPT-4o (OpenAI)	Image input: 30 panoramic radiographs with a standardized patient-style prompt.	English	30 anonymized panoramic radiographs from 2 private clinics in Spain (patient consent obtained)	Short, paragraph-form text interpretations of the panoramic radiographs regarding lower wisdom teeth	N/A	Although GPT-4o has shown promising performance in interpreting panoramic radiographs, its limitations in accuracy and occasional generation of incorrect information prevent its clinical use.
Radiology report generation and patient communication									
Mago and Sharma (2023)^28^	To evaluate the potential usefulness of GPT-3 in DMFR for identification of radiographic anatomical landmarks, and learning about pathologies and their radiographic features	Questionnaire-based evaluation using 80 queries	GPT-3 (OpenAI)	Text input only: Questions about anatomical landmarks, pathologies, and radiographic features	English	Queries developed by an oral and maxillofacial radiologist	Text-based answers to the queries	All questions and the Likert scale were provided in the main text, the answers from GPT were not provided.	ChatGPT is efficient and nearly accurate in describing the pathology, characteristic radiographic features, and describing anatomical landmarks.
Gao et al (2024)^27^	To propose and evaluate the multi-level objective alignment transformer network for generating fine-grained panoramic X-ray reports	Developing and testing a language model on a newly constructed dataset (562 image-report pairs)	MLAT incorporating a GPT2-Chinese-based decoder	Panoramic radiographs	Chinese and English	562 sets of panoramic radiographs and reports collected from Hangzhou Dental Hospital (Feb 2020 to Sep 2022), annotated by 13 dentists	Automatically generated fine-grained dental radiology reports describing tooth and disease findings at object-level granularity	N/A	MLAT significantly outperformed state-of-the-art baselines in generating fine-grained, accurate panoramic X-ray reports.
Stephan et al (2024)^29^	To assess the effectiveness of GPT in generating radiology reports from panoramic radiographs	Study assessing GPT’s outputs generated from structured checkbox lists completed by dental students	GPT-4.0 (OpenAI)	Text input: structured checkbox lists of diagnostic findings (derived from image analysis by students)	German	Two dental panoramic radiographs, collected from the Department of Oral and Maxillofacial Surgery at the University Medical Centre Mainz, Germany, with various pathologies	AI-generated radiology reports based solely on information from the completed checkbox lists	N/A	ChatGPT shows considerable potential for generating radiology reports, with high readability and error-free text, but currently lacks accuracy and completeness compared to student-generated reports.
Dasanayaka et al (2025)^26^	To develop and evaluate a multimodal AI system using LLMs for automated panoramic X-ray report generation and question-answering	Development and evaluation of a multimodal AI pipeline using curated and open datasets	Llama 3 8B (Meta), with domain adaptation and instruction fine-tuning; Blip-2 used for image captioning (vision-language model)	Images: panoramic radiographs Text: associated radiology captions, reports, and dental Q&A in Sinhala and English	English and Sinhala	4 datasets created: (1) Image and Caption Dataset (annotated by dental radiology experts), (2) Radiology Reports Dataset (curated by professionals), (3) Sinhala Language Corpus (web, subtitles, Wikipedia), (4) Sinhala Dental Q&A Dataset (from social media, curated by dentists)	(1) Automated radiology report for input image; (2) Chatbot-style responses to dental questions in Sinhala and English	Code available at https://github.com/ChirathD/Radiology-Report-Generation. Datasets are partly publicly available on Hugging Face	The multimodal AI system achieved 81.3% overall diagnostic accuracy. The Sinhala Q&A system enabled patient interaction in native language. Approach is promising for bridging dental service gaps in low-resource settings.

Abbreviations: AI = artificial intelligence; CBCT = cone-beam computed tomography; CT = computed tomography; DMFR = dento-maxillofacial radiology; FAQ = frequently asked question; LLM = large language model; MCQ = multiple-choice question; MLAT = multi-level objective alignment transformer; ÖSYM = Ölçme, Seçme ve Yerleştirme Merkezi; SAQ = short-answer question.

In terms of data sources, 8 studies used data from hospital or university archives, 5 used official guidelines, board examinations, or question banks, and the remaining studies created datasets by expert annotation or from open-access repositories. Model outputs included multiple-choice answers, diagnostic impressions, explanations, structured radiology reports, and patient education responses. The input and/or output language used in the studies included English, Chinese, German, Korean, Japanese, Turkish, and Sinhala. Table 3 exhibits the performance and evaluation methods of LLMs in the included studies. The performance of LLMs varied widely in diagnosing dental diseases and conditions, with accuracy ranging from 37% to 92.5%. Subjective expert ratings for differential diagnosis and treatment planning were between 3.6 and 4.7 on a 5-point scale. For DMFR-related qualification exams and board-style questions, LLMs’ accuracy ranged from 33.3% to 86.1%. For automated radiology report generation, reported accuracy ranged from 70.4% to 81.3%. Subjective expert scoring of report and answer quality ranged from 2.4 to 4.3 out of 5 or 6.1 to 7.5 out of 10. Regarding the evaluation approaches, 9 studies used subjective evaluation methods (such as Likert scales, rankings, or free-text assessment), 6 studies used objective evaluation methods (such as yes or no answer, 1 correct answer for MCQs), and the remaining combined both methods. 

**Table 3.** Performance and evaluation methods of LLMs in the included studies.

Author	Purpose of the study	Training, fine-tuning set	Test set	Ground truth	Output quality assessment	LLM performance	Qualification of the clinicians competed with LLM	Clinician performance
DMFR-related qualification exams and educational questions								
Jeong et al (2024)^13^	To evaluate the performance of 4 LLM-based chatbots compared to dental students in a DMFR examination	N/A	52 exam questions (16 basic knowledge, 27 imaging/equipment, 9 image interpretation; 38 MCQ, 14 SAQ)	Official answer keys from the radiology course instructors	Objective assessment: 1 correct answer for MCQs. Subjective assessment: SAQs assessed as correct or incorrect by radiologists, Answers that deviated from the standard or contained spelling errors were marked incorrect.	Accuracy rates: ChatGPT 50.0%, ChatGPT Plus 65.4%, Bard 50.0%, Bing Chat 63.5%. Highest chatbot accuracy was in basic knowledge (ChatGPT Plus 93.8%); all chatbots <35% in image interpretation. All chatbots <60% on MCQs, better on SAQs.	120 dental students (58 third-year, 62 fifth-year) who had completed relevant radiology coursework and exams	Overall accuracy: 81.2%. Accuracy by content: Basic knowledge: 78.7% Imaging/equipment: 83.5% Image interpretation: 78.5% MCQ: 80.5% SAQ: 82.9%
Morishita et al (2024)^15^	To assess the capabilities of GPT-4V with image recognition in answering image-based questions from the Japanese National Dental Examination	N/A	160 image-based questions (7 compulsory, 55 general, 98 clinical practical questions)	Official correct answers from the Ministry of Health, Labour and Welfare of Japan for the 116th National Dental Examination	Objective: 1 correct answer	Overall correctness rate = 35.0% Compulsory question = 57.1% General question = 43.6% Clinical practical question = 28.6% Dental radiology = 50%	N/A	N/A
Mohammad-Rahimi et al (2024)^14^	To evaluate the performance of 6 AI chatbots on controversial and difficult questions in oral pathology, oral medicine, and oral radiology, including the quality and validity of their citations	N/A	44 oral pathology/medicine questions and 22 oral radiology questions	Independent scoring by board-certified oral pathologists and radiologists (3 each) using a 5-point Likert scale	Subjective: expert panel grading of answer quality on a 5-point Likert scale	Mean score (Claude) (oral and maxillofacial pathology and medicine) = 4.34 ± 0.58 Mean score (GPT-4) (oral and maxillofacial pathology and medicine) = 4.29 ± 0.62 Mean score (Bing) (oral and maxillofacial pathology and medicine) = 3.45 ± 0.57 Mean score (GPT-4) (oral and maxillofacial radiology) = 3.62 ± 1.01 Mean score (Bing) (oral and maxillofacial radiology) = 2.38 ± 0.98	N/A	N/A
Hak-Sun and Gyu-Tae (2025)^11^	To evaluate and compare the quality of national dental board-style examination questions generated by a LLM and by human experts	N/A	40 multiple-choice questions (20 LLM-generated, 20 human-generated) covering oral/maxillofacial radiology, answered by 30 senior dental students	Two oral and maxillofacial radiologists	Objective: difficulty index, discrimination index, distractor efficiency, statistical comparison Subjective: students asked to guess question origin	Median (Difficulty index) (LLM) = 55.00 % Median (Discrimination index) (LLM) = 0.29 Median (Distractor efficiency) (LLM) = 80.00%	30 senior undergraduate dental students, who completed their first semester.	Median (difficulty index) (human) = 50.00% Median (discrimination index) (human) = 0.14 Median (distractor efficiency) (human) = 80.00%
Turunç Oğuzman and Yurdabakan (2024)^18^	To compare the performance of GPT-3.5 and Bard through multiple-choice dental specialty entrance examination questions	N/A	126 MCQs questions in prosthodontics and 123 MCQs questions in radiology in Turkish. MCQs	Official answer from ÖSYM for each question	Objective: 1 correct answer	ChatGPT: Correctness rate: 35.7% in prosthodontics and 52.8% in dentomaxillofacial radiology Bard: Correctness rate: 38.9% in prosthodontics and 52.8% in dento-maxillofacial radiology	N/A	N/A
Tassoker (2025)^17^	To evaluate and compare the performance of GPT-3.5, GPT-4 Omni (4o), Google Bard, and Microsoft Copilot in answering multiple-choice oral radiology questions from the dental specialty admission exam in Turkey	N/A	123 text-based, multiple-choice oral radiology questions	Official answers from the question bank	Objective: accuracy, word count, and response time	Accuracy (ChatGPT-4o) = 86.1% Accuracy (Google Bard) = 61.8% Accuracy (ChatGPT-3.5) = 43.9% Accuracy (Microsoft Copilot) = 41.5%	N/A	N/A
Russe et al (2024)^16^	To develop a content-aware chatbot based on GPT-3.5-Turbo and GPT-4 specialized on the German S2 CBCT dental imaging guideline and compare performance to humans	N/A	40 questions derived from consensus recommendations in the German S2 CBCT guideline prepared by an experience practitioner	Consensus recommendations from the German S2 CBCT guideline	Objective: accuracy of recommendations (Yes or No) and correctness of explanations (error-free vs inaccurate). Subjective: 5-point Likert scale ratings (explanation quality, content relevance, language appropriateness, trustworthiness).	GPT-4: 100% correct recommendations 87.5% correct explanations GPT-3.5-Turbo: 92.5% correct recommendations 57.5% correct explanations	Two early-career practitioners (first-/second-year training, CBCT guideline experience), 2 experienced practitioners (11/12 years’ experience, radiation protection certified)	Early-career: 77.5% and 87.5% correct answers Experienced: 100% correct answers
Helvacioglu-Yigit et al (2025)^12^	To compare the quality and readability of the responses generated by 3 AI chatbots in answering frequently asked questions related to oral and maxillofacial radiology to assess their suitability for patient education.	N/A	15 text-based DMFR FAQ questions	Evaluations by 3 board-certified DMFR specialists	Subjective assessment: responses were evaluated on scientific adequacy, ease of understanding, and overall reader satisfaction using a 7-point Likert scale, ranging from 1 (Strongly Disagree) to 4 (Neutral) to 7 (Strongly Agree)	Mean (scientific adequacy) (ChatGPT-3.5) = 4.8 Mean (scientific adequacy) (Gemini 1.5 Pro) = 4.2 Mean (scientific adequacy) (Copilot) = 4.3 Mean (ease of understanding) (ChatGPT-3.5) = 5.6 Mean (ease of understanding) (Gemini 1.5 Pro) = 3.9 Mean (ease of understanding) (Copilot) = 3.8 Mean (overall reader satisfaction) (ChatGPT-3.5) = 4.9 Mean (overall reader satisfaction) (Gemini 1.5 Pro) = 3.5 Mean (overall reader satisfaction) (Copilot) = 3.2	N/A	N/A
Diagnosis of dental diseases and conditions								
Uranbey et al (2024)^25^	To assess the diagnostic accuracy and therapeutic strategies of GPT in comparison to dental professionals across 12 clinical cases	N/A	Signs, symptoms, and radiographic findings retrieved from the records of 12 patients at Oral and Maxillofacial Surgery, Ordu University, Turkey	Evaluations by 30 attending physicians including 12 surgery and 18 radiology residents	Subjective: a 5-point scale	GPT differential diagnoses: median/mean score = 4/3.85 Treatment plans: median/mean score = 4/4.13	N/A	N/A
Hu et al (2024)^21^	To assess the performance of GPT in generating diagnoses based on chief complaint and CBCT radiologic findings	N/A	102 CBCT cases (48 dental diseases, 54 neoplastic/cystic diseases) with corresponding chief complaint and radiologic findings	Radiologic impressions and diagnoses by 2 experienced radiologists (10 and 15 years of experience); for neoplastic/cystic cases, postoperative pathological diagnoses	Subjective: 5-point Likert scale by 2 radiologists on: 1. Diagnostic accuracy (5 for all diagnosis is correct and 1 for all diagnosis is incorrect) 2. Completeness (5 for 80%-100% diagnoses are included and 1 for 0%-20% diagnoses are included) 3. Text quality (5 for no text error and 1 for more than 5 text errors) Objective: consistency with pathological diagnosis for neoplastic/cystic cases; error analysis of text outputs	Accuracy Dental diseases = 3.6/5 Neoplastic/cystic diseases = 3.8/5 Completeness Dental diseases= 4.6/5 Neoplastic/cystic diseases = 4.4/5 Text quality Dental diseases= 4.5/5 Neoplastic/cystic diseases = 4.7/5 ChatGPT's first diagnosis matched pathology in 38.9% of cases; 88.7% of text items were error-free	Radiologist A: 10 years of experience Radiologist B: 15 years of experience Radiologist E: 1 year of experience	For neoplastic/cystic cases: Radiologist A+B’s first diagnosis matched pathology in 48.1% of cases; Radiologist E’s first diagnosis matched pathology in 31.5% of cases
Silva et al (2024)^24^	To evaluate the performance of GPT in describing radiolucent lesions in panoramic radiographs and establishing differential diagnoses	N/A	28 panoramic radiographs, each with a single radiolucent lesion	Consensus descriptions and diagnoses by 3 experienced oral and maxillofacial radiologists	Subjective assessment: 3 rankings for all 3 categories	Accuracy Internal structure (radiodensity): 0.75 ± 0.44. Internal structure (loculation): 0.61 ± 0.50. Periphery (margin type): 0.93 ± 0.22. Periphery (cortication): 0.73 ± 0.29. Shape: 0.43 ± 0.50. Location (affected bone): 0.93 ± 0.26. Location (side): 0.68 ± 0.48. Location (region): 0.34 ± 0.39. Location (teeth/structures): 0.32 ± 0.46. Effect on adjacent structures: 0.22 ± 0.39. Origin: 0.87 ± 0.35. Behaviour: 0.87 ± 0.43. Nature: 0.50 ± 0.49.	N/A	N/A
Aşar et al (2025)^20^	To evaluate the accuracy of LLMs in detecting supernumerary teeth on periapical radiographs	CGPT-4V trained with 20 annotated periapical radiographs (10 with, 10 without supernumerary teeth)	180 periapical radiographs (90 with and 90 without supernumerary teeth)	Gold standard established by consensus of 3 experienced dentists for each radiograph	Subjective assessment using a 3-point Likert scale: Score 0: incorrect response; Score 1: partially correct response; Score 2: fully correct response.	Accuracy (GPT-4V) (fully correct with supernumerary teeth) = 37% Accuracy (GPT-4o) (fully correct with supernumerary teeth) = 55% Accuracy (CGPT-4V) (fully correct with supernumerary teeth) = 72%	N/A	N/A
Kahalian et al (2024)^22^	To evaluate the performance of GPT-4.0 in identifying anatomical landmarks, cysts, and tumours in oral and maxillofacial radiographic images, and to assess whether providing diagnostic clues improves accuracy	N/A	52 radiographic images (panoramic radiographies, periapical radiographies, cone-beam computed tomography sections)	Evaluations by 1 oral and maxillofacial radiologist and 1 anatomy specialist	Subjective: Prompt score measurement: 0: the prediagnosis of the anatomical or pathological condition was not in the pre-diagnosis list. 1: the prediagnosis of the anatomical or pathological condition was in the pre-diagnosis list. 2: the anatomical or pathological structure prediction was true or in the first place in the list of pre-diagnosis.	True prediagnosis rate without any clues = 30.7% True prediagnosis rate with at least 1 clue was included = 56.9% Average prompt score (anatomical structures) = 9.3 Average prompt score (odontogenic and non-odontogenic cyst) = 4.1 Average prompt score (odontogenic and non-odontogenic tumour) = 7.6	N/A	N/A
Salmanpour et al (2025)^23^	To evaluate the diagnostic accuracy of GPT-4.0 in determining the labiolingual position of impacted maxillary canines and detecting resorptive changes in adjacent incisors using panoramic radiographs	N/A	105 images for canine localization and 50 images (25 with and 25 without resorption) for resorption detection	Consensus of 3 orthodontists with at least 5 years of experience, based on panoramic and CBCT images (Fleiss’ kappa: 1.00 for localization, 0.814 for resorption)	Objective: Correct: ChatGPT-4.0 successfully identified the impacted canine(s) with the correct tooth or teeth Incorrect: ChatGPT-4.0 did not identified the impacted canine(s)	Overall accuracy (determining the labiolingual localization) = 37.1% Weighted average (precision) = 33.6%	N/A	N/A
Ana et al (2025)^19^	To evaluate the accuracy and reproducibility of GPT-4o in interpreting panoramic radiographs for lower third molar assessment	N/A	30 panoramic radiographs, each with 30 repeated ChatGPT-4o responses (900 total responses)	Grading by 2 experts in oral surgery	Subjective assessment using a 3-point Likert scale: A score of 2 was assigned to correct answers, 1 to partially correct or incomplete answers, and 0 to incorrect answers. In cases of disagreement between the raters, a third expert (A.S.) acted as arbitrator to resolve the discrepancy	Accuracy: 92.5 4% (95% CI: 35.27%-41.62%) Percent agreement among repeated responses: 82.7%	N/A	N/A
Radiology report generation and patient communication								
Mago et al (2023)^28^	To evaluate the potential usefulness of GPT-3 in DMFR for identification of radiographic anatomical landmarks, and learning about pathologies and their radiographic features	N/A	80 questions including 19 anatomical landmarks, 31 oral/maxillofacial pathologies, 30 radiographic features	Evaluations by an experienced oral and maxillofacial radiologist	Subjective assessment using 4-point scale: (4) The application responded with adequate information such as mentioning the characteristic features or explaining the basic pathophysiology; (3) The application responded, however, did not provide adequate information. For instance, a 1-line answer which does not describe any characteristic feature; (2) The application did not know the response to the question; (1) The application led to error messages.	Mean score Anatomical landmarks = 3.94 Oral-maxillofacial pathologies = 3.85 Radiographic features of oral and maxillofacial pathologies = 3.96	N/A	N/A
Gao et al (2024)^27^	To propose and evaluate the multi-level objective alignment transformer network for generating fine-grained panoramic X-ray reports	450 training image-report pairs, with data augmentation to 3600 pairs	112 image-report pairs (19.93% of the dataset) from the collected X-ray images and reports	Reports written and annotated by 13 experienced dentists for each image	Objective: BLEU (1-4), Meteor, Rouge-L, BERTScore, and clinical efficacy metrics (accuracy, precision, recall, F1)	MLAT achieved highest scores across all evaluation metrics (BLEU-1: 50.11, BLEU-4: 21.69, Meteor: 26.34, Rouge-L: 51.30, BERTScore: 0.7813) Clinical efficacy: Accuracy = 70.4% Recall = 55.7% F1-score = 0.554	N/A	N/A
Stephan et al (2024)^29^	To assess the effectiveness of GPT in generating radiology reports from panoramic radiographs	N/A	Each of 100 dental students analysed 2 images, producing both written reports and checkbox lists for AI input (total number of reports: 100 AI-generated, 100 student-written)	Reference reports manually created by a senior physician with extensive clinical experience in dental radiology using a standardized template, based on the same checkbox lists	Objective: The readability and complexity of both student-written and AI-generated texts were assessed using the Flesch reading ease (FRE) score and the Lesbarhetsindex (LIX) readability index. Text similarity was evaluated by using the BERT score.	Mean score (FRE) (ChatGPT) = 50.55 SD (FRE) (ChatGPT) = 7.80 Mean score (LIX) (ChatGPT with semester 1) = 48.81 SD (LIX) (ChatGPT with semester 1) = 3.44 Mean score (LIX) (ChatGPT with semester 3) = 48.01 SD (LIX) (ChatGPT with semester 3) = 2.84	A total of 100 dental students from all 5 clinical semesters participated in the study with the following distribution across semesters—semester 1: *n* = 20, semester 2: *n* = 19, semester 3: *n* = 21, semester 4: *n* = 20, and semester 5: *n* = 20	Mean score (FRE) (student) = 51.19 SD (FRE) (student) = 5.02 Mean score (LIX) (semesters 1) = 46.27 SD (LIX) (semesters 1) = 4.0 Mean score (LIX) (semesters 3) = 51.64 SD (LIX) (semester 3) = 4.89
Dasanayaka et al (2025)^26^	To develop and evaluate a multimodal AI system using LLMs for automated panoramic X-ray report generation and question-answering	Llama 3 8B: instruct fine-tuned with 1200 radiology reports and continued pre-trained on 12.3B-token Sinhala corpus. Blip-2: fine-tuned on 1000 image-caption pairs. Q&A: 2500 Sinhala dental Q&A pairs	Blip-2: 100 image-caption pairs. Llama 3 8B: 200 radiology reports. Q&A: 1000 Sinhala dental MCQs	Expert-annotated captions and reports (for image/report generation), and consensus answers from certified dental professionals (for Q&A)	Subjective assessment for radiology report generation: weighted average score given by dental professionals Subjective assessment for Sinhala language Q&A in the dental domain: weighted average score given by dental professionals	Diagnostic accuracy Overall: 81.3% Dental caries: 87.9% Impacted teeth: 89.7% Bone loss: 88% Periapical lesions: 81.8% Report quality: 7.5/10 Q&A performance: Accuracy 74.1% Answer quality: 6.1/10	N/A	N/A

Abbreviations: DMFR = dento-maxillofacial radiology; LLM = large language model; MCQ = multiple-choice question; MLAT = multi-level objective alignment transformer; SAQ = short-answer question; SD = standard deviation.

The evaluation of the risk of bias in the included studies was conducted across 3 domains, including dataset, performance evaluation, and reference standard, using a customized assessment criterion that was adapted from the TRIPOD-LLM reporting guideline.^10^ Regarding concerns related to the dataset, 7 studies were rated as “high” risk of bias mainly due to insufficient detail about the origin, selection, or time period of the images or textual data used. For performance evaluation, 3 studies were rated as “high” or “unclear” due to use of a single human assessor, inadequate descriptions of the number and qualifications of assessors, or lack of clarity in the evaluation process. Regarding the reference standard, 6 studies were rated as “high” or “unclear” risk of bias as the qualification of the examiners or the process for establishing the reference standard was not adequately specified or only a single assessor was used. The risk of bias assessment for all included studies is presented in Table S2.

## Discussion

This study aimed to systematically review the current performance of LLMs in the DMFR field. Since 2023, a growing number of studies have investigated the performance of widely available LLMs for various DMFR-related tasks. The included studies investigated the use of LLMs for tasks including the production and answering of DMFR-related qualification exams and educational questions, diagnosis and treatment recommendations, and radiology report generation and patient communication.

### DMFR-related qualification exams and educational questions

There is increasing interest in investigating the performance of LLMs in qualification exams.^13^^,^^15^ Several studies have evaluated LLMs’ abilities to answer examination questions across various dental disciplines and languages. Jeong et al^13^ compared ChatGPT Plus, Bard, and Bing Chat to dental students in a Korean dental school setting. The results revealed an overall correctness rate of 81.2% for dental students compared to a 65.4% rate for ChatGPT Plus. ChatGPT Plus outperformed students in basic knowledge questions (93.8% vs 78.7%), but students excelled in radiographic image interpretation (78.5% vs 33.3%). Morishita et al^15^ found that GPT-4V achieved an overall correctness rate of 35% on the Japanese national dental examination with its highest performance in compulsory questions (57.1%) and 50% in dental radiology. In the Turkish dental specialty entrance examination, it was observed that GPT-3.5 and Bard performed better in DMFR questions (52.8%) than in prosthodontics (35.7-38.9%).^18^ Mohammad-Rahimi et al^14^ compared 6 AI chatbots on challenging questions in oral pathology, oral medicine, and oral radiology and found that GPT-4 achieved the highest average score (4.07/5) though its performance in radiology (3.62) was lower than in other domains. These findings suggest that question complexity from dental school exams, licensing exams, to specialty training, can significantly affect LLM performance. Notably, AI chatbots seem to face more challenges in DMFR-related and image-based questions compared to fundamental dental knowledge. Russe et al^16^ reported that while GPT-3.5-Turbo and GPT-4 performed well on MCQs regarding CBCT recommendations (correctness rates of 92.5% and 100%), their accuracy in providing explanatory textual descriptions was relatively lower (57.5% and 87.5%). In addition to answering exam questions, recent studies have explored the ability of LLMs to generate high-quality board-style exam questions. Hak-Sun and Gyu-Tae^11^ reported that GPT-4o could create DMFR board-style questions comparable in quality to those written by radiologists. However, it is noteworthy that 3 out of 44 questions created by ChatGPT were based on hallucinations. This highlights the necessity of expert review to ensure the accuracy and reliability of AI-generated educational materials.

### LLM-assisted diagnosis of dental diseases and conditions

Detection of pathologies and identification of anatomical landmarks have also been investigated as potential applications for LLMs in DMFR.^30^ Uranbey et al^25^ reported that GPT-3.5 achieved favourable accuracy in generating differential diagnoses (mean 3.85/5) and treatment plans (4.13/5) based on patients’ clinical signs, symptoms, and radiographic findings. These results exhibit GPT-3.5’s promising capability in deducing potential diagnoses and formulating reasonable treatment strategies. Similarly, Hu et al^21^ evaluated the performance of GPT-3.5 in generating diagnostic impressions from patient chief complaints and CBCT radiological findings. Their results showed that while GPT-3.5 could generate comprehensive and well-structured diagnostic text outputs, its performance varied depending on disease type and diagnostic complexity, and occasionally produced hallucinated or imprecise statements. Some studies investigated LLMs’ abilities to analyse radiographic images using structured prompts. Silva et al^24^ found that GPT-3.5 achieved correctness rates ranging from 0.73 to 0.93 when describing various radiographic features, including origin, location, periphery, internal structure, and behaviour of the pathologies, on panoramic radiographs. However, GPT-3.5 faced challenges in recognizing the exact shape of pathologies, accurately locating teeth and anatomical features, and evaluating the effect of pathologies on adjacent structures. Other studies explored the use of LLMs for detecting specific radiographic features. Aşar et al^20^ reported that a customized GPT-4V achieved a diagnostic accuracy of 91% in identifying supernumerary teeth on periapical radiographs. In contrast, Salmanpour and Akpınar^23^ found that GPT-4.0 achieved a lower diagnostic accuracy of 37% when detecting impacted canines and root resorption on panoramic radiographs. Kahalian et al^22^ evaluated GPT-4.0’s ability to recognize anatomical and pathological structures on periapical, panoramic, and CBCT images, with and without additional clues regarding location, internal structure, peripheral structure, and adjacent structures. Their results showed that GPT-4.0 tended to misdiagnose closely located anatomical structures, but its performance improved with the inclusion of supplementary information, though its capacity to distinguish among a diverse range of differential diagnoses remained limited.

### AI-assisted radiology report generation and patient communication

The use of LLMs and multimodal AI systems for automated radiology report generation has been explored with great interest. Recently, an increasing number of studies have focused on developing multimodal LLMs to automate the generation of CT and MRI radiology reports or produce radiologic impressions that are both professionally and linguistically suitable across a wide range of radiology examinations.^31–33^ Mago and Sharma^28^ evaluated the performance of GPT-3 in describing anatomical landmarks and radiographic features of pathologies in the oral and maxillofacial region. Their findings demonstrated that GPT-3 achieved high accuracy in identifying radiographic anatomical landmarks but its responses regarding pathological features tended to lack in-depth detail. Notably, GPT-3 provided more accurate and detailed answers when questions were specifically and comprehensively phrased. Gao et al^27^ developed a multi-level objective alignment transformer model to generate reports for panoramic radiographs that provided detailed descriptions of dental diseases and conditions at the tooth level. While this model achieved a promising overall accuracy of 0.7, its ability to detect and describe jaw pathologies remains unknown. Stephan et al^29^ evaluated GPT-4’s ability to generate reports for panoramic radiographs based on diagnostic checkbox lists completed by dental students. Their results showed that GPT-4-generated reports exhibited a high degree of textual similarity to reference reports but contained significantly fewer findings than student-written reports. GPT-4’s reports were concise and free from spelling, grammar, or punctuation errors whereas student reports were more detailed but contained such errors. Importantly, no hallucinations were found in GPT-4’s reports as the reports were generated solely from information provided in the checkbox list. Dasanayaka et al^26^ developed a multimodal AI system combining computer vision and LLMs for automated generation of radiology reports for panoramic radiographs and answering patient queries in English and Sinhala. Their system achieved an overall diagnostic accuracy of 81.3% for the generated reports with a quality score of 7.5/10, and a question-answering accuracy of 74.1% with a quality score of 6.1. These studies suggest that LLMs and multimodal AI systems have the potential to automate the generation of clinically relevant and detailed radiology reports from dental radiographic images. Such AI tools might help to reduce clinician workload, standardize reporting, and improve diagnostic efficiency particularly in public hospital settings and resource-limited regions.

### Prospects and challenges of LLMs in DMFR

The integration of the image recognition capabilities in LLMs represents a significant advancement in AI applications for radiology. This innovation has expanded the role of AI chatbots from solely text-based interactions to include the interpretation of diagnostic images and the generation of radiology reports. Recent studies have demonstrated that LLMs are increasingly capable of supporting a range of DMFR tasks, including answering and producing board-style examination questions,^11^^,^^15^^,^^18^ assisting with diagnosis and clinical decision-making,^20^^,^^21^^,^^25^ generating structured radiology reports,^26–29^ and patient communication.^26^ However, the performance of LLMs in DMFR remains inconsistent, particularly when applied to complex diagnostic tasks and image-based questions. In some cases, LLMs may generate hallucinated pathological descriptions, and thus, it is necessary for clinicians and researchers to carefully review AI-generated outputs.^11^^,^^21^ Additionally, it is important to note that the ground truth in some studies used for comparison with LLM outputs was established by human assessors of unspecified qualifications.^19^^,^^26^^,^^27^^,^^29^ Ideally, high-quality image-text datasets should be created with ground truth established by multiple assessors, including at least 1 radiologist, to ensure validity and reliability. The development of standardized, multidisciplinary databases that integrate radiological, clinical, and patient-reported information is crucial for enhancing both the training and validation of LLMs and multimodal AI systems. Some other issues regarding data privacy and security require careful consideration.^10^ The use of patient medical images and sensitive clinical information requires strict adherence to ethical standards and data protection regulations. Researchers and clinicians must thoroughly review the terms of service, data handling practices, and privacy safeguards of any LLM platform before utilizing it in research or clinical practice to ensure compliance with ethical and legal requirements. Despite these challenges, LLMs have great potential as valuable tools to assist clinicians with pathology localization, lesion feature assessment, treatment strategy formulation, rapid documentation of findings, and improved patient communication.

This systematic review has several limitations. First, there is currently no widely accepted or validated quality assessment tool specifically designed for evaluating studies on LLMs in radiology. In this review, the customized risk-of-bias assessment criteria adapted from the TRIPOD-LLM reporting guideline were used, focusing on the clarity of dataset origin, the robustness of performance evaluation methods, and the validity of the reference standards.^10^ While these domains were selected to address major reliability concerns, the effectiveness of the customized criteria in identifying potential risk of bias in LLM studies within DMFR remains uncertain. Second, quantitative synthesis or meta-analysis was not performed due to considerable heterogeneity in the objectives, methodologies, and outcome measures of the included studies. Despite these limitations, this systematic review provided a comprehensive overview of the current performance of LLMs in DMFR, highlighting the need for standardized methodologies, validated quality assessment tools, and robust reference standards in future studies. Future studies should also investigate the cost-effectiveness of implementing LLMs in diagnostic workflows, patient management, educational tasks as well as their capabilities in formulating recommendations to assist both patients and clinicians with DMFR-related tasks.

## Conclusion

Based on the current evidence, the following conclusions could be drawn:

- Recently developed LLMs have shown promising potential in supporting a range of DMFR tasks, including the production and answering of DMFR-related qualification exams and educational questions, diagnosis and treatment recommendations, and radiology report generation and patient communication.
- Despite encouraging results, publicly accessible AI chatbots currently demonstrate lower and more varied accuracy in DMFR-specific tasks, especially in complex diagnostic and image-based cases.
- The overall consistency, completeness, and clarity of LLM-generated outputs are not yet sufficient for unsupervised clinical application. Expert review remains necessary, particularly given the risk of hallucinated responses.

## Supplementary Material

twaf060_Supplementary_Data

## References

[1] Yang R , Tan TF, Lu W, Thirunavukarasu AJ, Ting DSW, Liu N. Large language models in health care: development, applications, and challenges. Health Care Sci. 2023;2:255-263. 10.1002/hcs2.61 38939520 PMC11080827

[2] Wang J , Zhang G, Wang W, Zhang K, Sheng Y. Cloud-based intelligent self-diagnosis and department recommendation service using Chinese medical BERT. J Cloud Comp. 2021;10:4. 10.1186/s13677-020-00218-2

[3] Su MC , Lin LE, Lin LH, Chen YC. Assessing question characteristic influences on ChatGPT’s performance and response-explanation consistency: insights from Taiwan’s Nursing Licensing Exam. Int J Nurs Stud. 2024;153:104717. 10.1016/j.ijnurstu.2024.104717 38401366

[4] Le Guellec B , Lefèvre A, Geay C, et al Performance of an open-source large language model in extracting information from free-text radiology reports. Radiol Artif Intell. 2024;6:e230364. 10.1148/ryai.230364 38717292 PMC11294959

[5] Liu S , Wright AP, Patterson BL, et al Assessing the value of ChatGPT for clinical decision support optimization. medRxiv: 2023.02.21.23286254, 2023. 10.1101/2023.02.21.23286254

[6] Hirosawa T , Harada Y, Yokose M, Sakamoto T, Kawamura R, Shimizu T. Diagnostic accuracy of differential-diagnosis lists generated by generative pretrained transformer 3 chatbot for clinical vignettes with common chief complaints: a pilot study. Int J Environ Res Public Health. 2023;20:3378. 36834073 10.3390/ijerph20043378 PMC9967747

[7] Jeblick K , Schachtner B, Dexl J, et al ChatGPT makes medicine easy to swallow: an exploratory case study on simplified radiology reports. Eur Radiol. 2024;34:2817-2825. 10.1007/s00330-023-10213-1 37794249 PMC11126432

[8] Siepmann R , Huppertz M, Rastkhiz A, et al The virtual reference radiologist: comprehensive AI assistance for clinical image reading and interpretation. Eur Radiol. 2024;34:6652-6666. 10.1007/s00330-024-10727-2 38627289 PMC11399201

[9] Moher D , Liberati A, Tetzlaff J, Altman DG, PRISMA Group. Preferred reporting items for systematic reviews and meta-analyses: the PRISMA statement. Ann Intern Med. 2009;151:264-269, W64. 19622511 10.7326/0003-4819-151-4-200908180-00135

[10] Gallifant J , Afshar M, Ameen S, et al The TRIPOD-LLM reporting guideline for studies using large language models. Nat Med. 2025;31:60-69. 10.1038/s41591-024-03425-5 39779929 PMC12104976

[11] Hak-Sun K , Gyu-Tae K. Can a large language model create acceptable dental board-style examination questions? A cross-sectional prospective study Can a large language model create acceptable dental board-style examination questions? A cross-sectional prospective study. J Dent Sci. 2025;20:895-900. 40224064 10.1016/j.jds.2024.08.020 PMC11993092

[12] Helvacioglu-Yigit D , Demirturk H, Ali K, Tamimi D, Koenig L, Almashraqi A. Evaluating artificial intelligence chatbots for patient education in oral and maxillofacial radiology. Oral Surg Oral Med Oral Pathol Oral Radiol. 2025;139:750-759. 10.1016/j.oooo.2025.01.001 40044548

[13] Jeong H , Han S-S, Yu Y, Kim S, Jeon KJ. How well do large language model-based chatbots perform in oral and maxillofacial radiology? Dentomaxillofac Radiol. 2024;53:390-395. 10.1093/dmfr/twae021 38848473 PMC11358622

[14] Mohammad-Rahimi H , Khoury ZH, Alamdari MI, et al Performance of AI chatbots on controversial topics in oral medicine, pathology, and radiology. Oral Surg Oral Med Oral Pathol Oral Radiol. 2024;137:508-514. 10.1016/j.oooo.2024.01.015 38553304

[15] Morishita M , Fukuda H, Muraoka K, et al Evaluating GPT-4V’s performance in the Japanese national dental examination: a challenge explored. J Dent Sci. 2024;19:1595-1600. 10.1016/j.jds.2023.12.007 39035269 PMC11259620

[16] Russe MF , Rau A, Ermer MA, et al A content-aware chatbot based on GPT 4 provides trustworthy recommendations for cone-beam CT guidelines in dental imaging. Dentomaxillofac Radiol. 2024;53:109-114. 10.1093/dmfr/twad015 38180877 PMC11003655

[17] Tassoker M. ChatGPT-4 Omni’s superiority in answering multiple-choice oral radiology questions. BMC Oral Health. 2025;25:173-178. 10.1186/s12903-025-05554-w 39893407 PMC11786404

[18] Turunç Oğuzman R , Yurdabakan ZZ, Department of Prosthodontics, Faculty of Dentistry, Altınbaş University, Istanbul, Turkey. Performance of chat generative pretrained transformer and bard on the questions asked in the dental specialty entrance examination in Turkey regarding Bloom’s revised taxonomy. Curr Res Dent Sci. 2024;34:25-34. 10.5152/CRDS.2024.23261

[19] Ana S , Stefania A, Alberto Herranz C, Ana Isabel Castillo V, Victor Diaz-Flores G, Yolanda F. Decoding wisdom: evaluating ChatGPT's accuracy and reproducibility in analyzing orthopantomographic images for third molar assessment decoding wisdom: evaluating ChatGPT’s accuracy and reproducibility in analyzing orthopantomographic images for third molar assessment. Comput Struct Biotechnol J. 2025;28:141-147. 40271108 10.1016/j.csbj.2025.04.010 PMC12017887

[20] Aşar EM , İpek İ, Bi Lge K. Customized GPT-4V(ision) for radiographic diagnosis: can large language model detect supernumerary teeth? BMC Oral Health. 2025;25:756. 10.1186/s12903-025-06163-3 40399904 PMC12096622

[21] Hu Y , Hu Z, Liu W, et al Exploring the potential of ChatGPT as an adjunct for generating diagnosis based on chief complaint and cone beam CT radiologic findings. BMC Med Inform Decis Mak. 2024;24:55. 10.1186/s12911-024-02445-y 38374067 PMC10875853

[22] Kahalian S , Rajabzadeh M, Öçbe M, Medisoglu MS. ChatGPT-4.0 in oral and maxillofacial radiology: prediction of anatomical and pathological conditions from radiographic images. Folia Med (Plovdiv). 2024;66:863-868. 10.3897/folmed.66.e135584 39774357

[23] Salmanpour F , Akpınar M. Performance of chat generative pretrained transformer-4.0 in determining labiolingual localization of maxillary impacted canine and presence of resorption in incisors through panoramic radiographs: a retrospective study. Am J Orthod Dentofacial Orthop. 2025;168:220-231. 10.1016/j.ajodo.2025.02.017 40208160

[24] Silva TP , Andrade-Bortoletto MFS, Ocampo TSC, et al Performance of a commercially available generative pre-trained transformer (GPT) in describing radiolucent lesions in panoramic radiographs and establishing differential diagnoses. Clin Oral Investig. 2024;28:204. 10.1007/s00784-024-05587-5 PMC10924032 38459362

[25] Uranbey Ö , Özbey F, Kaygısız Ö, Ayrancı F. Assessing ChatGPT’s diagnostic accuracy and therapeutic strategies in oral pathologies: a cross-sectional study. Curēus (Palo Alto, CA). 2024;16:e58607. 10.7759/cureus.58607 PMC11102887 38770501

[26] Dasanayaka C , Dandeniya K, Dissanayake MB, Gunasena C, Jayasinghe R. Multimodal AI and large language models for orthopantomography radiology report generation and Q&A. ASI. 2025;8:39. 10.3390/asi8020039

[27] Gao N , Yao R, Liang R, Chen P, Liu T, Dang Y. Multi-level objective alignment transformer for fine-grained oral panoramic X-ray report generation. IEEE Trans Multimedia. 2024;26:7462-7474. 10.1109/TMM.2024.3368922

[28] Mago J , Sharma M. The potential usefulness of ChatGPT in oral and maxillofacial radiology. Curēus (Palo Alto, CA). 2023;15:e42133. 10.7759/cureus.42133 PMC10355343 37476297

[29] Stephan D , Bertsch A, Burwinkel M, et al AI in dental radiology-improving the efficiency of reporting with ChatGPT: comparative study. J Med Internet Res. 2024;26:e60684. 39714078 10.2196/60684 PMC11704643

[30] Sohrabniya F , Hassanzadeh-Samani S, Ourang SA, et al Exploring a decade of deep learning in dentistry: a comprehensive mapping review. Clin Oral Investig. 2025;29:143. 10.1007/s00784-025-06216-5 39969623

[31] Voinea ȘV , Mămuleanu M, Teică RV, Florescu LM, Selișteanu D, Gheonea IA. GPT-driven radiology report generation with fine-tuned Llama 3. Bioengineering (Basel). 2024;11:1043. 10.3390/bioengineering11101043 PMC11504957 39451418

[32] Li C-Y , Chang K-J, Yang C-F, et al Towards a holistic framework for multimodal LLM in 3D brain CT radiology report generation. Nat Commun. 2025;16:2258. 10.1038/s41467-025-57426-0 40050277 PMC11885477

[33] Zhang L , Liu M, Wang L, et al Constructing a large language model to generate impressions from findings in radiology reports. Radiology. 2024;312:e240885. 10.1148/radiol.240885 39287525

