# Supplementary material for: The performance of large language models in dentomaxillofacial radiology: a systematic review
Source: Dentomaxillofac Radiol. 2025 Aug 12;54(8):613–31. doi: 10.1093/dmfr/twaf060 (PMC12653761; doi:10.1093/dmfr/twaf060)
Supplement: twaf060_Supplementary_Data [file twaf060_supplementary_data.zip › Revised Supplementary materials.docx]

**Supplementary materials**

**Supplementary Table S1.** exhibits the specific search strategies used for all databases

| **Search on 6th June 2025** | **Search strategy** |
| --- | --- |
| PubMed  (Record number = 353) | ***Search strategy:***  Group 1 (with ORs): AI/LLM/Chatbot related terms.  Group 2 (with ORs): Dentomaxillofacial radiology-related terms.  AND: Combines the two groups, so only articles containing at least one term from each group are retrieved.  ***Search Query:***  ((natural language processing) OR (NLP) OR (language model) OR (chatbot) OR (chatgpt) OR (GPT) OR (BARD) OR (BERT) OR (BING)) **AND** ((dentomaxillofacial radiology) OR (oral radiology) OR (dental radiology) OR (DMFR) OR (OMFR) OR (cone beam computed tomography) OR (CBCT) OR (panoramic radiography) OR (OPG) OR (periapical radiography) OR (cephalometric radiography) OR (dental x-ray))  ***PubMed Search Translation:***  ("natural language processing"[MeSH Terms] OR ("natural"[All Fields] AND "language"[All Fields] AND "processing"[All Fields]) OR "natural language processing"[All Fields] OR "NLP"[All Fields] OR (("language"[MeSH Terms] OR "language"[All Fields] OR "languages"[All Fields] OR "language s"[All Fields] OR "programming languages"[MeSH Terms] OR ("programming"[All Fields] AND "languages"[All Fields]) OR "programming languages"[All Fields]) AND ("model"[All Fields] OR "model s"[All Fields] OR "modeled"[All Fields] OR "modeler"[All Fields] OR "modeler s"[All Fields] OR "modelers"[All Fields] OR "modeling"[All Fields] OR "modelings"[All Fields] OR "modelization"[All Fields] OR "modelizations"[All Fields] OR "modelize"[All Fields] OR "modelized"[All Fields] OR "modelled"[All Fields] OR "modeller"[All Fields] OR "modellers"[All Fields] OR "modelling"[All Fields] OR "modellings"[All Fields] OR "models"[All Fields])) OR ("generative artificial intelligence"[MeSH Terms] OR ("generative"[All Fields] AND "artificial"[All Fields] AND "intelligence"[All Fields]) OR "generative artificial intelligence"[All Fields] OR "chatbot"[All Fields]) OR ("generative artificial intelligence"[MeSH Terms] OR ("generative"[All Fields] AND "artificial"[All Fields] AND "intelligence"[All Fields]) OR "generative artificial intelligence"[All Fields] OR "chatgpt"[All Fields]) OR "GPT"[All Fields] OR "BARD"[All Fields] OR "BERT"[All Fields] OR "BING"[All Fields]) AND ("dentomaxillofac radiol"[Journal] OR ("dentomaxillofacial"[All Fields] AND "radiology"[All Fields]) OR "dentomaxillofacial radiology"[All Fields] OR ("oral radiol"[Journal] OR ("oral"[All Fields] AND "radiology"[All Fields]) OR "oral radiology"[All Fields]) OR (("dental health services"[MeSH Terms] OR ("dental"[All Fields] AND "health"[All Fields] AND "services"[All Fields]) OR "dental health services"[All Fields] OR "dental"[All Fields] OR "dentally"[All Fields] OR "dentals"[All Fields]) AND ("radiology"[MeSH Terms] OR "radiology"[All Fields] OR "radiography"[MeSH Terms] OR "radiography"[All Fields] OR "radiology s"[All Fields])) OR ("dentomaxillofac radiol"[Journal] OR "dmfr"[All Fields]) OR "OMFR"[All Fields] OR ("cone beam computed tomography"[MeSH Terms] OR ("cone beam"[All Fields] AND "computed"[All Fields] AND "tomography"[All Fields]) OR "cone beam computed tomography"[All Fields] OR ("cone"[All Fields] AND "beam"[All Fields] AND "computed"[All Fields] AND "tomography"[All Fields]) OR "cone beam computed tomography"[All Fields]) OR "CBCT"[All Fields] OR ("radiography, panoramic"[MeSH Terms] OR ("radiography"[All Fields] AND "panoramic"[All Fields]) OR "panoramic radiography"[All Fields] OR ("panoramic"[All Fields] AND "radiography"[All Fields])) OR "OPG"[All Fields] OR (("periapical"[All Fields] OR "periapically"[All Fields] OR "periapicals"[All Fields]) AND ("diagnostic imaging"[MeSH Subheading] OR ("diagnostic"[All Fields] AND "imaging"[All Fields]) OR "diagnostic imaging"[All Fields] OR "radiography"[All Fields] OR "radiography"[MeSH Terms] OR "radiographies"[All Fields] OR "radiographys"[All Fields])) OR (("cephalometric"[All Fields] OR "cephalometrical"[All Fields] OR "cephalometrically"[All Fields] OR "cephalometrics"[All Fields]) AND ("diagnostic imaging"[MeSH Subheading] OR ("diagnostic"[All Fields] AND "imaging"[All Fields]) OR "diagnostic imaging"[All Fields] OR "radiography"[All Fields] OR "radiography"[MeSH Terms] OR "radiographies"[All Fields] OR "radiographys"[All Fields])) OR ("radiography, dental"[MeSH Terms] OR ("radiography"[All Fields] AND "dental"[All Fields]) OR "dental radiography"[All Fields] OR "dental x ray"[All Fields]))  ***Individual Term Translations:***  natural language processing: "natural language processing"[MeSH Terms] OR ("natural"[All Fields] AND "language"[All Fields] AND "processing"[All Fields]) OR "natural language processing"[All Fields]  language: "language"[MeSH Terms] OR "language"[All Fields] OR "languages"[All Fields] OR "language's"[All Fields] OR "programming languages"[MeSH Terms] OR ("programming"[All Fields] AND "languages"[All Fields]) OR "programming languages"[All Fields]  model: "model"[All Fields] OR "model's"[All Fields] OR "modeled"[All Fields] OR "modeler"[All Fields] OR "modeler's"[All Fields] OR "modelers"[All Fields] OR "modeling"[All Fields] OR "modelings"[All Fields] OR "modelization"[All Fields] OR "modelizations"[All Fields] OR "modelize"[All Fields] OR "modelized"[All Fields] OR "modelled"[All Fields] OR "modeller"[All Fields] OR "modellers"[All Fields] OR "modelling"[All Fields] OR "modellings"[All Fields] OR "models"[All Fields]  chatbot: "generative artificial intelligence"[MeSH Terms] OR ("generative"[All Fields] AND "artificial"[All Fields] AND "intelligence"[All Fields]) OR "generative artificial intelligence"[All Fields] OR "chatbot"[All Fields]  chatgpt: "generative artificial intelligence"[MeSH Terms] OR ("generative"[All Fields] AND "artificial"[All Fields] AND "intelligence"[All Fields]) OR "generative artificial intelligence"[All Fields] OR "chatgpt"[All Fields]  dentomaxillofacial radiology: "Dentomaxillofac Radiol"[Journal:__jid7609576] OR ("dentomaxillofacial"[All Fields] AND "radiology"[All Fields]) OR "dentomaxillofacial radiology"[All Fields]  oral radiology: "Oral Radiol"[Journal:__jid8806621] OR ("oral"[All Fields] AND "radiology"[All Fields]) OR "oral radiology"[All Fields]  dental: "dental health services"[MeSH Terms] OR ("dental"[All Fields] AND "health"[All Fields] AND "services"[All Fields]) OR "dental health services"[All Fields] OR "dental"[All Fields] OR "dentally"[All Fields] OR "dentals"[All Fields]  radiology: "radiology"[MeSH Terms] OR "radiology"[All Fields] OR "radiography"[MeSH Terms] OR "radiography"[All Fields] OR "radiology's"[All Fields]  DMFR: "Dentomaxillofac Radiol"[Journal:__jid7609576] OR "dmfr"[All Fields]  cone beam computed tomography: "cone-beam computed tomography"[MeSH Terms] OR ("cone-beam"[All Fields] AND "computed"[All Fields] AND "tomography"[All Fields]) OR "cone-beam computed tomography"[All Fields] OR ("cone"[All Fields] AND "beam"[All Fields] AND "computed"[All Fields] AND "tomography"[All Fields]) OR "cone beam computed tomography"[All Fields]  panoramic radiography: "radiography, panoramic"[MeSH Terms] OR ("radiography"[All Fields] AND "panoramic"[All Fields]) OR "panoramic radiography"[All Fields] OR ("panoramic"[All Fields] AND "radiography"[All Fields])  periapical: "periapical"[All Fields] OR "periapically"[All Fields] OR "periapicals"[All Fields]  radiography: "diagnostic imaging"[Subheading] OR ("diagnostic"[All Fields] AND "imaging"[All Fields]) OR "diagnostic imaging"[All Fields] OR "radiography"[All Fields] OR "radiography"[MeSH Terms] OR "radiographies"[All Fields] OR "radiographys"[All Fields]  cephalometric: "cephalometric"[All Fields] OR "cephalometrical"[All Fields] OR "cephalometrically"[All Fields] OR "cephalometrics"[All Fields]  radiography: "diagnostic imaging"[Subheading] OR ("diagnostic"[All Fields] AND "imaging"[All Fields]) OR "diagnostic imaging"[All Fields] OR "radiography"[All Fields] OR "radiography"[MeSH Terms] OR "radiographies"[All Fields] OR "radiographys"[All Fields]  dental x-ray: "radiography, dental"[MeSH Terms] OR ("radiography"[All Fields] AND "dental"[All Fields]) OR "dental radiography"[All Fields] OR "dental x ray"[All Fields] |
| [Medline (via Ovid)](https://eproxy.lib.hku.hk/login?url=http://gateway.ovid.com/ovidweb.cgi?T=JS&MODE=ovid&PAGE=main&NEWS=n&DBC=y&D=mesz)  (Record number = 269) | ((natural language processing) OR (NLP) OR (language model) OR (chatbot) OR (chatgpt) OR (GPT) OR (BARD) OR (BERT) OR (BING)) AND ((dentomaxillofacial radiology) OR (oral radiology) OR (dental radiology) OR (DMFR) OR (OMFR) OR (cone beam computed tomography) OR (CBCT) OR (panoramic radiography) OR (OPG) OR (periapical radiography) OR (cephalometric radiography) OR (dental x-ray))  Ovid MEDLINE(R) ALL <1946 to June 06, 2025>  1 (natural language processing or NLP or language model or chatbot or chatgpt or GPT or BARD or BERT or BING).af. 190137  2 (dentomaxillofacial radiology or oral radiology or dental radiology or DMFR or OMFR or cone beam computed tomography or CBCT or panoramic radiography or OPG or periapical radiography or cephalometric radiography or dental x-ray).af. 49551  3 1 and 2 269  4 from 3 keep 1-269 269 |
| [Embase](https://eproxy.lib.hku.hk/login?url=http://gateway.ovid.com/ovidweb.cgi?T=JS&MODE=ovid&PAGE=main&NEWS=n&DBC=y&D=mesz) (via Ovid)  (Record number = 382) | ((natural language processing) OR (NLP) OR (language model) OR (chatbot) OR (chatgpt) OR (GPT) OR (BARD) OR (BERT) OR (BING)) AND ((dentomaxillofacial radiology) OR (oral radiology) OR (dental radiology) OR (DMFR) OR (OMFR) OR (cone beam computed tomography) OR (CBCT) OR (panoramic radiography) OR (OPG) OR (periapical radiography) OR (cephalometric radiography) OR (dental x-ray))  Embase <1974 to 2025 June 06>  1 (natural language processing or NLP or language model or chatbot or chatgpt or GPT or BARD or BERT or BING).af. 176186  2 (dentomaxillofacial radiology or oral radiology or dental radiology or DMFR or OMFR or cone beam computed tomography or CBCT or panoramic radiography or OPG or periapical radiography or cephalometric radiography or dental x-ray).af. 74746  3 1 and 2 382 |
| Web of Science  (Record number = 531) | (ALL=(natural language processing) OR ALL=(NLP) OR ALL=(language model) OR ALL=(chatbot) OR ALL=(chatgpt) OR ALL=(GPT) OR ALL=(BARD) OR ALL=(BERT) OR ALL=(BING)) AND (ALL=(dentomaxillofacial radiology) OR ALL=(oral radiology) OR ALL=(dental radiology) OR ALL=(DMFR) OR ALL=(OMFR) OR ALL=(cone beam computed tomography) OR ALL=(CBCT) OR ALL=(panoramic radiography) OR ALL= (OPG) OR ALL=(periapical radiography) OR ALL=(cephalometric radiography) OR ALL=(dental x-ray)) |
| Scopus  (Record number = 86) | ( ALL ( "natural language processing" ) OR ALL ( "nlp" ) OR TITLE-ABS-KEY ( "language model" ) OR TITLE-ABS-KEY ( "chatbot" ) OR TITLE-ABS-KEY ( "chatgpt" ) OR TITLE-ABS-KEY ( "gpt" ) OR TITLE-ABS-KEY ( "bard" ) OR TITLE-ABS-KEY ( "bert" ) OR TITLE-ABS-KEY ( "bing" ) AND TITLE-ABS-KEY ( "dentomaxillofacial radiology" ) OR TITLE-ABS-KEY ( "oral radiology" ) OR TITLE-ABS-KEY ( "dental radiology" ) OR TITLE-ABS-KEY ( "dmfr" ) OR TITLE-ABS-KEY ( "omfr" ) OR TITLE-ABS-KEY ( "cone beam computed tomography" ) OR TITLE-ABS-KEY ( "cbct" ) OR TITLE-ABS-KEY ( "panoramic radiography" ) OR TITLE-ABS-KEY ( "opg" ) OR TITLE-ABS-KEY ( "periapical radiography" ) OR TITLE-ABS-KEY ( "cephalometric radiography" ) OR TITLE-ABS-KEY ( "dental x-ray" ) ) |

**Supplementary Table S2.** Risk-of-bias assessment scores of the included studies according to customized criteria adapted from the TRIPOD-LLM evaluating three domains: “dataset”, “performance evaluation”, and “reference standard”. Each domain was assessed for concerns regarding the risk of bias in methodological quality and rated on a 3-point scale reflecting these concerns as low (+), high (-), or unclear (?)

|  | **Dataset** | **Performance evaluation** | **Reference standard** |
| --- | --- | --- | --- |
| Russe et al. (2024) [16] | Low | Low | **Unclear** due to lack of confirmation regarding the involvement of a radiologist |
| Uranbey et al. (2024) [25] | **Unclear** due to insufficient details regarding language and case period | Low | Low |
| Hu et al. (2024) [21] | Low | Low | Low |
| Morishita et al. (2024) [15] | Low | Low | Low |
| Jeong et al. (2024) [13] | Low | Low | Low |
| Silva et al. (2024) [24] | Low | Low | Low |
| Mohammad-Rahimi et al. (2024) [14] | **High** due to insufficient details regarding the origin and creation time of the textual dataset used for chatbot evaluation. | Low | Low |
| Turunç Oğuzman et al. (2024) [18] | Low | Low | Low |
| Mago et al. (2024) [28] | **High** due to insufficient details regarding time of creation and process for question selection | **High** due to only one human assessor | **High** due to **the reference standard being established by only a single assessor** |
| Gao et al. (2024) [27] | **High** due to lack of detail on how images were selected | **High** due to insufficiently detailed descriptions of the performance evaluation process | **Unclear** due to unknown qualification of the dental practitioners. |
| Stephan et al. (2024) [29] | **High** due to lack of detail on how images were selected | **Unclear** due to unclear **number and qualifications of assessors**for subjective evaluations | **High** due to **the reference standard being established by only a single assessor** and unknown qualification of the assessor |
| Hak-Sun et al. (2025) [11] | Low | Low | Low |
| Tassoker et al. (2025) [17] | Low | Low | Low |
| Kahalian et al. (2024) [22] | **High** due to lack of detail on how images were selected | Low | Low |
| Aşar et al. (2025) [20] | Low | Low | Low |
| Ana et al. (2025) [19] | Low | Low | **Unclear** due to unknown qualification of the experts in oral surgery. |
| Helvacioglu-Yigit et al. (2025) [12] | Low | Low | Low |
| Dasanayaka et al. (2025) [26] | **High** due to lack of detail on how images were selected | Low | **Unclear** due to unknown qualification of the medical professionals. |
| Salmanpour et al. (2025) [23] | Low | Low | Low |
